# Real‐World Outcomes of Newly Diagnosed Multiple Myeloma Patients Treated Before the Era of Anti‐CD38 Antibodies: The EMMY Cohort From 2017 to 2020

**DOI:** 10.1002/cam4.70619

**Published:** 2025-03-14

**Authors:** Laure Vincent, Olivier Decaux, Aurore Perrot, Bruno Royer, Thomas Chalopin, Arthur Bobin, Margaret Macro, Denis Caillot, Lionel Karlin, Caroline Jacquet, Cécile Sonntag, Mohamad Mohty, Laurent Frenzel, Arnaud Jaccard, Salomon Manier, Laurence Sanhes, Driss Chaoui, Philippe Moreau, Ronan Garlantézec, Nathalie Texier, Chanaz Louni, Zakaria Maarouf, Herve Avet Loiseau, Cyrille Hulin, Karim Belhadj Merzoug

**Affiliations:** ^1^ Department of Clinical Hematology Montpellier University Hospital Center Montpellier France; ^2^ Centre Hospitalier Universitaire de Rennes ‐ Hôpital Pontchaillou Rennes France; ^3^ Centre Hospitalier Universitaire de Toulouse IUCT‐Oncopole, Université de Toulouse, UPS, Service d'Hématologie Toulouse France; ^4^ Saint Louis Hospital Paris France; ^5^ Centre hospitalier universitaire de Tours, Hospital Tours France; ^6^ CHU Poitiers Poitiers France; ^7^ IHBN—Centre Hospitalier Universitaire Caen France; ^8^ Hématologie Clinique Centre Hospitalier Universitaire de Dijon Bourgogne Dijon France; ^9^ Hôpital Lyon Sud Hospices Civils de Lyon Pierre‐Bénite France; ^10^ CHU de Nancy Vandoeuvre Les Nancy Vandoeuvre‐les‐Nancy France; ^11^ University Hospital Hôpital Hautepierre Strasbourg France; ^12^ Department of Haematology Saint Antoine Hospital Paris France; ^13^ Department of Haematology Institut Necker Paris France; ^14^ Referral Center for AL Amyloidosis Limoges France; ^15^ Hematologie clinique Centre Hospitalier Universitaire de Limoges Limoges France; ^16^ Hématologie Centre Hospitalier Universitaire de Lille Lille France; ^17^ Centre Hospitalier Perpignan Perpignan France; ^18^ Hôpital Victor Dupouy Argenteuil France; ^19^ Department of Haematology Centre Hospitalier Universitaire de Nantes Nantes France; ^20^ Santé publique et épidémiologie Centre Hospitalier Universitaire de Rennes Rennes France; ^21^ Kappa Santé Paris France; ^22^ IFM Paris France; ^23^ IUCT‐Oncopole Toulouse Toulouse France; ^24^ Centre Hospitalier Universitaire de Bordeaux Pessac Cedex France; ^25^ Unité Hémopathies Lymphoïdes Centre Hospitalier Universitaire Henri Mondor Créteil France

**Keywords:** ASCT patients, first‐line therapy, multiple myeloma, NTE patients, real world

## Abstract

**Aims/Background:**

Recent agents have profoundly reshaped the multiple myeloma (MM) landscape. Their real‐world impacts need to be assessed over the long term.

**Methods:**

EMMY is a non‐interventional, prospective dynamic cohort, conducted in France, since 2017, with 900 patients enrolled each year. Newly diagnosed MM (NDMM) who initiated a treatment from 2017 to 2020 are here described.

**Results:**

A total of 1036 non‐transplant eligible (NTE) patients (median age: 74.9 years) and 561 patients who received autologous stem cell transplantation (ASCT) (median age: 60.6 years) were enrolled. For ASCT patients, a shift in induction treatment from bortezomib‐thalidomide‐dexamethasone (VTd) (29.1%) to bortezomib‐lenalidomide‐dexamethasone (VRd) (55.1%) marked the period. Maintenance treatment with R after ASCT became a standard (75% of patients). In NTE patients, R‐based regimens were increasingly used from 29.4% in 2017 (of whom Rd.: 17.0%, VRd: 10.6%) to 73.3% in 2020 (of whom Rd.: 21.8%, VRd: 48.5%). Median progression‐free survival (mPFS) was 46.5 months (95% CI: 37.8–50.6) and 18.7 months (95% CI: 16.3–20.8) in ASCT and NTE patients, respectively. In the ASCT group, patients treated with and without R maintenance had a mPFS of 51.8 (95% CI: 44.1–NA) and 29.6 months (95% CI: 21.8–40.9), respectively. In the NTE group, the mPFS was 26.3 (95% CI: 21.9–30.9) and 14.6 months (95% CI: 11.9–17.7) in patients who received an R‐based and non‐R‐based regimen, respectively. The estimated 48‐month overall survival rates were 89% (95% CI: 84.5–92.2) and 63% (95% CI: 58.5–67.1) for ASCT and NTE patients, respectively.

**Conclusions:**

The 2017–2020 period was marked by the expansion of R use in both NDMM ASCT and NTE patients.

## Graphical abstract

A total of 1036 non‐transplant eligible (NTE) patients and 561 patients who received autologous stem cell transplantation (ASCT) were enrolled in Emmy. In the ASCT group, patients treated with and without R maintenance had a mPFS of 51.8 (95% CI: 44.1–NA) and 29.6 months (95% CI: 21.8–40.9), respectively. In the NTE group, the mPFS was 26.3 (95% CI: 21.9–30.9) and 14.6 months (95% CI: 11.9–17.7) in patients who received an R‐based and non‐R‐based regimen, respectively. The 2017–2020 period was marked by the expansion of R use in both NMDD ASCT and NTE patients.

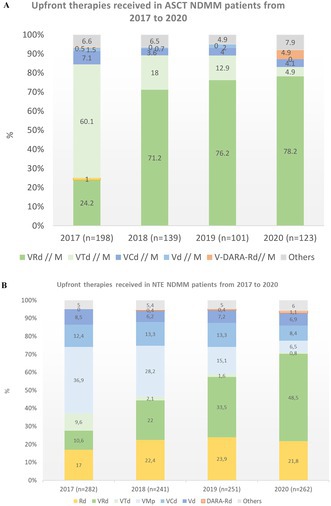

## 1 Introduction

Multiple myeloma (MM) is the second most common haematologic malignancy. It accounts for 1% of all cancers and approximately 10% of haematological malignancies, with a European incidence estimated at 4.5–6.0/100,000 people per year [1, 2, 3, 4, 5]. With a median age at diagnosis of 70 years [6], MM is a disease that mainly affects older people. While the survival rate of patients with MM has increased considerably over the past 20 years, only a minority (10%–15%) of patients achieve the expected survival rate for the corresponding general population [7, 8].

Improved survival for patients with MM has been attributed to the emergence of several pivotal therapies over the last few decades. In the 90s, in patients with newly diagnosed MM (NDMM), the introduction of high‐dose melphalan plus autologous stem cell transplantation (ASCT) has significantly enhanced the median overall survival (mOS) of the youngest patients [9]. New agents, such as proteasome inhibitors (PIs) (e.g., bortezomib) and immunomodulatory drugs (IMiDs) (e.g., thalidomide, lenalidomide [R]), have gradually become the standard of care for NDMM through clinical evidence of their efficacy, and approval in Europe [10, 11, 12, 13]. The widespread use of the anti‐CD38 therapy (daratumumab [Dara]) is the latest paradigm shift in NDMM following results from recent large phase III clinical studies [14, 15, 16, 17, 18].

In line with these therapeutic advances, the EU guidelines recommended at the time of the study period, triple combined bortezomib‐thalidomide‐dexamethasone (VTd) or bortezomib‐lenalidomide‐dexamethasone (VRd) followed by high‐dose therapy with ASCT and lenalidomide maintenance as standard therapy for ‘fit’ patients aged < 71 years without comorbidities ([2, 5, 19]). In non‐transplant‐eligible (NTE) patients with NDMM, bortezomib‐melphalan and prednisone (VMp) was the only standard of care until the arrival of R with the options of Rd. for all patients and VRd for fitter patients [5, 20, 21].

Most therapies have been assessed in pivotal clinical trials involving highly selected and standardised patients who do not reflect typical day‐to‐day patients with MM [22, 23, 24]. Older age, functional decline, comorbidities such as renal failure, previous exposure to certain drugs, are all factors that keep patients out of clinical trials [5, 24]. Real‐world studies provide supplemental evidence and complement the data for these patients. In MM, several international and more local studies initiated in recent years have mostly concerned relapsed patients. Most studies have completed enrolment with no possibility of assessing the use, benefit and tolerance of current therapeutic developments for patients with NDMM [25, 26, 27, 28, 29, 30].

In this context, the Epidemiology of Multiple MYeloma (EMMY) study, designed to enrol the entire cohort of patients with MM who initiate any line of treatment in a large variety of MM‐treating sites, presents an opportunity to assess the real‐world therapeutic management of MM patients in France over the long term [31, 32, 33]. In EMMY, approximately 40% of patients were enrolled at the time of initiating upfront treatment. Here, we present the results of NDMM patients for the first 4 years of inclusion (2017–2020).

## 2 Patients and Methods

### 2.1 Patients and Study Design

EMMY is a real‐life, non‐interventional, longitudinal, historical cohort study conducted in France in 75 MM specialist sites that are all members of the French‐speaking Intergroupe Francophone du Myélome (IFM) and are representative of the 118 IFM French sites including general hospitals, university hospitals and private hospitals and clinics. The EMMY protocol has been detailed in a dedicated recent publication [34].

Patient recruitment is dynamic; the comprehensiveness of patients diagnosed with MM initiating a new line of treatment over a 3‐month reference period (last quarter of the year) is identified each year at the participating sites. Patients enrolled in interventional clinical trials and those with non‐symptomatic MM are excluded. Patients are enrolled only once in the cohort and cannot be included again in subsequent years. The study plans to recruit at least 5000 patients in annual cohorts of 800–900, beginning in 2017 and continuing over at least 6 years.

Patients are retrospectively included in the cohort after they have been informed using written letter, and have not objected to the data collection. Their medical records are updated annually until the end of the study or death, whichever comes first.

The study is carried out in compliance with all relevant regulations, the Declaration of Helsinki and the applicable guidelines on Good Practices in Epidemiology and conducted in compliance with the General Data Protection Regulation and local regulations. According to local regulations, the study is recorded in France in the Health Data Hub registry (No. F20220518161845).

### 2.2 Objectives and Assessments

The primary objective of the study is to describe the treatment regimens received by patients and the changes in practice over time. The secondary objectives are to assess the real‐life efficacy of the different therapeutic combinations in terms of time to next treatment (TTNT), progression‐free survival (PFS), OS and response rates.

### 2.3 Data Collection

Data collection is carried out once a year at all participating sites. Recording of every line of treatment once the patients are enrolled comprises description of induction, intensification, ASCT, consolidation and maintenance, with initial dosage of the regimens and any changes in treatment. Therapies prior to patient recruitment are historically recorded in the database to enable a complete description of therapeutic patterns. Patient‐ and disease‐related characteristics are collected at the initiation of new treatment and biological tests, treatment response, progression, treatment discontinuation, together with reasons for discontinuation throughout the treatment lines.

Patient cytogenetic abnormalities, Eastern Cooperative Oncology Group‐Performance Status (ECOG‐PS) and International Staging System (ISS) stage are collected at the initiation of new treatment when patients are routinely assessed in a real‐world setting. Comorbidities are estimated using Charlson's Comorbidity Index (CCI), and frailty using the simplified frailty score based on age, CCI and ECOG‐PS [35]. Refractoriness to treatment differs slightly from the International Myeloma Working Group (IMWG) criteria [36] to match real‐life data more closely and comprises patients whose line of treatment discontinued within 6 months from the start without a partial response (PR) or better (primary refractoriness), and patients who initiated a subsequent line of treatment within 60 days after treatment discontinuation (secondary refractoriness).

### 2.4 Statistical Analysis

Data analyses are performed once a year. Patients are analysed by treatment line: those with NDMM and relapsed/refractory MM are analysed separately. The present publication targets only patients with NDMM and describes frontline treatment. Patient were included between 2017 and 2020. Patients with NDMM who received frontline therapy with ASCT (ASCT group) are described separately from NTE patients with NDMM (NTE group). R‐based first‐line treatments were any combinations that included lenalidomide (R) at the initiation of the line of treatment and comprised VRd. For ASCT patients, only induction treatments before transplantation were considered. V‐based regimens were any treatment that included bortezomib without lenalidomide (during the induction for ASCT patients).

Statistical analyses were conducted using SAS version 9.4 (SAS Institute Inc., Cary, NC, USA). Qualitative data are summarised by frequencies and percentages. Quantitative data are summarised by means with standard deviations or medians with 95% confidence intervals (CIs). Time‐to‐event analyses (OS, PFS, progression after the next line of therapy (PFS2) or TTNT), defined in the publication dedicated to the protocol, are performed using the Kaplan–Meier method. The results are reported as medians with the associated 95% CIs and presented as Kaplan–Meier survival curves. The significance level for analysis is set at 5% unless otherwise specified.

Data analyses investigated the impact of age (< 80 versus [vs.] ≥ 80 years in NTE patients and < 65 vs. ≥ 65 years in patients who received ASCT), frailty (frail vs. non‐frail), exposure to R (R‐ and non‐R‐based regimens), renal failure based on creatinine clearance at initiation (> 30 vs. ≤ 30 mL/min), comorbidities (CCI) and cytogenetic abnormalities at baseline (standard risk (SR) vs. high risk (HR) defined as del(17p) and/or t(4;14)).

The analyses and exploitation of the database were supervised by a scientific committee consisting of experts in MM and in epidemiology.

## 3 Results

In total, 3616 patients were included over the 2017–2020 period, including 1597 (44%) with NDMM. Of these, 1036 were in the NTE group (64.9%) (282 in 2017, 241 in 2018, 251 in 2019 and 262 in 2020) and 561 in the ASCT group (35.1%) (198 in 2017, 139 in 2018, 101 in 2019 and 123 in 2020). Patients were recruited evenly throughout France by university hospitals (57.2%), general hospitals (34.1%) and private clinics (8.6%), comprising 23.3% in Ile de France (*n* = 371), 22.3% in the North‐East (*n* = 356), 21% in the North‐West (*n* = 332), 20.2% in the South‐East (*n* = 322) and 13.5% in the South‐West and French overseas departments (*n* = 216). Patients had a median follow‐up of 23.7 months (interquartile range [IQR]: 12.3–39.0 months).

### 3.1 Patients Who Received ASCT

#### 3.1.1 Patients' Characteristics

Patients who received ASCT had a median age of 60.6 years, 13% were < 50 years of age when they initiated first‐line treatment and 28.2% (*n* = 158) were ≥ 65 years of age (Table 1). The patients were considered fit: 86.7% had ECOG‐PS 0 or 1, 76.5% had no relevant comorbidities and very few were considered frail at baseline (17.0%).

**TABLE 1:** Patient demographics and disease characteristics at baseline.

	NDMM ASCT (*n* = 561)	NDMM NTE (*n* = 1036)	NDMM Total (*n* = 1597)
Age (years)			
Median	60.6	74.9	69.8
IQR	55–65.5	69.6–81.5	62.3–77.8
< 50	73 (13%)	14 (1.4%)	87 (5.4%)
[50–55]	68 (12.1%)	14 (1.4%)	82 (5.1%)
[55–60]	129 (23%)	43 (4.2%)	172 (10.8%)
[60–65]	133 (23.7%)	55 (5.3%)	188 (11.8%)
[65–70]	129 (23%)	150 (14.5%)	279 (17.5%)
[70–75]	28 (5%)	247 (23.8%)	275 (17.2%)
[75–80]	1 (0.2%)	198 (19.1%)	199 (12.5%)
≥ 80	0 (0%)	315 (30.4%)	315 (19.7%)
Gender, *n* (%)			
Male	338 (60.2%)	526 (50.8%)	864 (54.1%)
Female	223 (39.8%)	510 (49.2%)	733 (45.9%)
ECOG‐PS, *n* (%)	436	740	1176
0–1	378 (86.7%)	491 (66.4%)	869 (73.9%)
≥ 2	58 (13.3%)	249 (33.6%)	307 (26.1%)
Comorbidities, *n* (%)			
0	429 (76.5%)	548 (52.9%)	977 (61.2%)
1	105 (18.7%)	279 (26.9%)	384 (24%)
≥ 2	27 (4.8%)	209 (20.2%)	236 (14.8%)
Charlson's Comorbidity Index total score			
0	477 (85%)	667 (64.4%)	1144 (71.6%)
1–2	76 (13.5%)	279 (26.9%)	355 (22.2%)
3–4	6 (1.1%)	61 (5.9%)	67 (4.2%)
≥ 5	2 (0.4%)	29 (2.8%)	31 (1.9%)
Frailty, *n* (%)	436	832	1268
Yes	74 (17.0%)	548 (65.9%)	622 (49.1%)
Year of first‐line initiation			
2017	198 (35.2%)	282 (27.2%)	480 (30.0%)
2018	139 (24.8%)	241 (23.3%)	380 (23.9%)
2019	101 (18.0%)	251 (24.2%)	352 (22.0%)
2020	123 (21.9%)	262 (25.3%)	385 (24.1%)
M protein type, *n* (%)			
IgG	301 (53.7%)	599 (57.8%)	900 (56.4%)
IgA	115 (20.5%)	216 (20.8%)	331 (20.7%)
Light chain	136 (24.2%)	200 (19.3%)	336 (21.0%)
Other	9 (1.6%)	21 (2.0%)	30 (1.9%)
Renal clearance (< 30 mL/min), *n* (%)	520	909	1429
Yes	18 (3.5%)	118 (13.0%)	136 (9.5%)
ISS, *n* (%)	360	555	915
Stage I	127 (35.3%)	139 (25.0%)	266 (29.1%)
Stage II	122 (33.9%)	164 (29.6%)	286 (31.3%)
Stage III	111 (30.8%)	252 (45.4%)	363 (39.7%)
Cytogenetic test, *n* (%)	473	830	1303
Yes	358 (75.7%)	468 (56.4%)	826 (63.4%)
Cytogenetic feature, *n* (%)	358	468	826
HR t(4;14) or del(17p)	70 (19.6%)	77 (16.4%)	147 (17.8%)
SR	288 (80.4%)	391 (83.5%)	679 (82.2%)
CRAB criteria, *n* (%)	544	1013	1557
At least one	489 (89.9%)	931 (91.9%)	1420 (91.2%)
Hypercalcaemia	101 (23.0%)	257 (25.4%)	358 (23.0%)
Renal impairment	95 (17.5%)	330 (32.6%)	425 (27.3%)
Anaemia	217 (39.9%)	521 (51.4%)	738 (47.4%)
Bone lesion	414 (76.0%)	648 (63.8%)	1062 (68.2%)
Type of sites, *n* (%)	561	1036	1597
CHU	345 (61.5%)	569 (54.9%)	914 (57.2%)
CH‐CHG	168 (29.9%)	377 (36.4%)	545 (34.1%)
Private clinic	48 (8.6%)	90 (8.7%)	138 (8.6%)

Abbreviations: ASCT: autologous stem cell transplant; CH‐CHG: hospital centre and general hospital centre; CHU: university hospital centre; CRAB: hypercalcaemia, renal failure, anaemia, and bone lesions; ECOG‐PS: Eastern Cooperative Oncology Group‐Performance Status; HR: high risk; IQR: interquartile range; ISS: International staging system; NDMM: newly diagnosed multiple myeloma; NTE: non‐transplant eligible; SR: standard risk.

Some patients (*n* = 18; 3.5%) received ASCT even though they had severe renal impairment at the start of initial treatment (glomerular filtration rate < 30 mL/min). Significantly more patients who received ASCT had ISS Stage III (84.6%) (*p* < 0.001). No patients had ISS Stage I. Other characteristics, including age, gender, ECOG‐PS, comorbidities and cytogenetic risk were similar between both groups (Table S1).

In routine practice, cytogenetic tests were carried out for 75.7% of patients who received ASCT with no identified differences in patient or site characteristics between those who were and were not tested. Test performance remained stable over the recruitment period, with 139 (79.9%), 86 (74.1%), 57 (70.4%) and 76 (74.5%) patients undergoing cytogenetic tests in 2017, 2018, 2019 and 2020, respectively. In total, 70 (19.6%) of the patients who received ASCT showed HR abnormalities (t(4;14) or del(17p)), and these patients accounted for 47.6% (70/147) of all HR patients with NDMM (Table S2).

#### 3.1.2 Therapeutic Management

**FIGURE 1:**
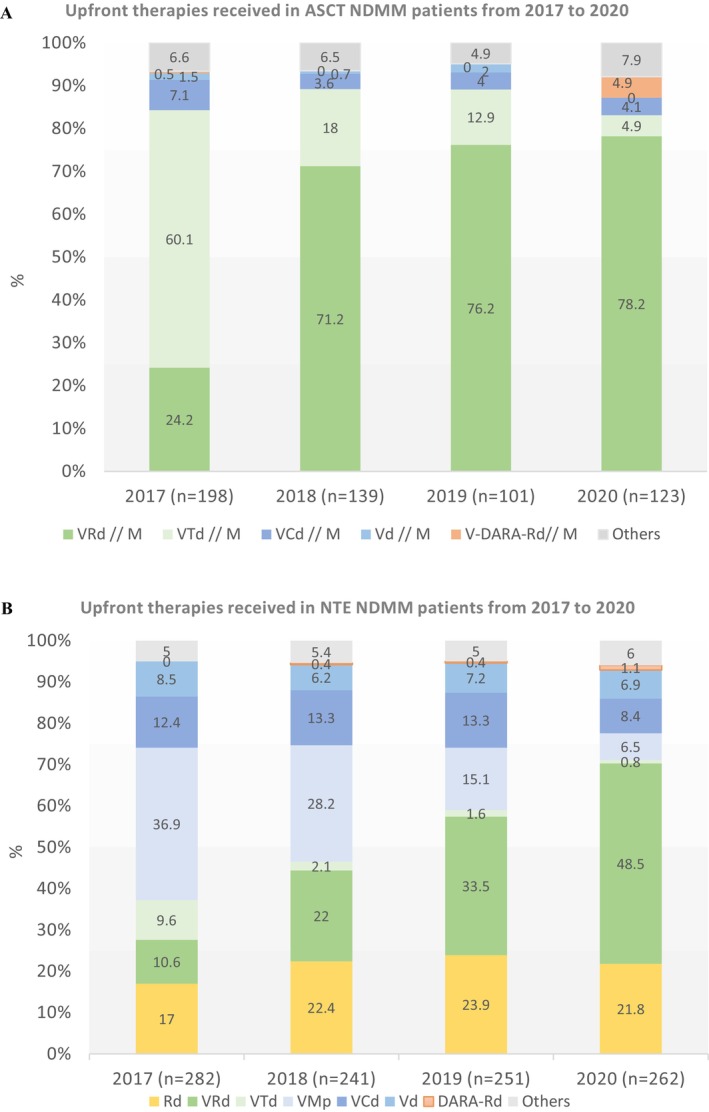
Combination therapy received in (A) patients with NDMM who received ASCT and (B) NDMM NTE patients with NDMM according to the year of treatment initiation. ASCT: autologous stem cell transplant; C: cyclophosphamide; d: dexamethasone; Dara: daratumumab; M: Melphalan; NDMM: newly diagnosed multiple myeloma; NTE: non‐transplant eligible; p: prednisolone; R: lenalidomide; T: Thalidomide; V: bortezomib.

The period 2017–2020 was marked by a shift in the therapeutic management of patients who received ASCT, characterised by a switch in the induction treatment from VTd (*n* = 163, 29.1% of patients who received ASCT) to VRd (*n* = 309, 55.1%) (Table 2, Figure 1A). VTd, which accounted for 60.1% of induction treatments in 2017, decreased to 18% in 2018 and 4.9% in 2020, whereas VRd increased from 24.2% in 2017 to 71.2%–78.2% over the 2018–2020 period (Figure 1A). Out of the 472 patients treated with VRd and VTd, 328 patients received consolidation therapy after transplantation, 69.3% and 69.9% in the VRd and VTd groups respectively.

**TABLE 2:** Agents and combination treatments.

	NDMM ASCT (*n* = 561)	NDMM NTE (*n* = 1036)
ASCT		
0	0	1036 (100%)
1	508 (90.6%)	
2	53 (9.5%)	
Proteasome inhibitor	558 (99.5%)	785 (75.8%)
Bortezomib	558 (99.5%)	784 (75.7%)
Carfilzomib	0 (0.0%)	1 (0.1%)
IMiD	524 (93.4%)	586 (56.6%)
Lenalidomide	346 (61.7%)	539 (52%)
Thalidomide	179 (31.9%)	45 (4.3%)
Pomalidomide	1 (0.2%)	4 (0.4%)
Anti‐CD38	11 (2%)	15 (1.4%)
Daratumumab	11 (2%)	14 (1.4%)
Isatuximab	0 (0.0%)	1 (0.1%)
Alkylator	549 (97.9%)	389 (37.5%)
Melphalan	542 (96.6%)	245 (23.6%)
Cyclophosphamide	57 (10.2%)	142 (13.7%)
Bendamustine	1 (0.2%)	5 (0.5%)
Other cytotoxic	2 (0.4%)	5 (0.5%)
Doxorubicin	2 (0.4%)	3 (0.3%)
Other	0 (0.0%)	2 (0.2%)
Most prevalent combinations		
First	VRd//M: 309 (55.1%)	VRd: 294 (28.4%)
Second	VTd//M: 163 (29.1%)	VMp: 227 (21.9%)
Third	VCd//: 28 (5%)	Rd: 219 (21.1%)
Fourth	VCRd//M: 11 (2%)	VCd: 123 (11.9%)
Fifth	V‐DaraRd//M: 7 (1.2%)	Vd: 75 (7.2%)
Treatment discontinuation		
Still ongoing	273 (48.7%)	326 (31.5%)
Discontinuation due to progression	68 (12.1%)	264 (25.5%)
Discontinuation due to AE	42 (7.5%)	151 (14.6%)
Discontinuation as per protocol	165 (29.4%)	193 (18.6%)
Unspecified reason	13 (2.3%)	102 (9.8%)
Primary refractory	2 (0.4%)	161 (15.6%)
Subsequent line started	130 (23.2%)	430 (41.5%)

Abbreviations: Anti‐CD38: anti‐CD38 therapy; ASCT: autologous stem cell transplant; C: cyclophosphamide; d: dexamethasone; Dara: daratumumab; IMiD: immunomodulatory drugs; M: melphalan; NDMM: newly diagnosed multiple myeloma; NTE: non‐transplant eligible; p: prednisolone; R: lenalidomide; T: thalidomide; V/bortezomib.

Treatment with R maintenance following ASCT was increasingly used over the years, including 62.6% of patients in 2017 and over 80% since 2019. Overall, 75% of ASCT patients received maintenance (*n* = 415). 60.1% of patients (98/163) received R maintenance after VTd induction and 83.2% of patients, after VRd induction (257/309).

IMiD‐based regimens were less often retained for patients with significant renal impairment (61.1% vs. 94.5%) who primarily received combinations with cyclophosphamide (C) (55.6% vs. 8.7%). C continued to be used in 10.2% (*n* = 57) of patients who received ASCT, with equal proportions over time ranging from 8.1% to 12.9%. HR ASCT patients received similar treatment to non‐HR ASCT patients (VRd 47.1% and 56.2%, VTd 32.9% and 28.5%, respectively) (Table S3).

Conversely, the 73 patients aged < 50 years more often received VRd (*n* = 45, 61.1% vs. *n* = 264, 54.1%) than VTd (*n* = 14, 19.2% vs. *n* = 149, 30.5%) compared with patients aged ≥ 50 years.

Among the ASCT group, 53 (9.5%) patients received a double transplantation. Recourse to this therapeutic option remained limited and stable over time. Median age was (58.9 years vs. 60.7 years) for patients with a single transplantation; of these, 9 patients aged < 50 years received a double transplantation (9/73, 12.3% < 50 years vs. 44/488, 9% ≥ 50 years). Cytogenetic testing was performed for most of these patients (87.5% vs. 74.4%, *p* = 0.04). Double transplant was preferred for patients with HR abnormalities (38.1% vs. 17.1%, *p* < 0.01) (Tables S4 and S5). Among the 70 HR ASCT patients, 16 (23%) received a double transplant. Induction with VRd (64.2% vs. 54.1%) or a quadruple combination comprising VR‐Dara‐d (4 patients received Dara out of reimbursement) or VRC‐d (13.5% vs. 2.2%) was favoured for those patients.

### 3.2 NTE Patients

#### 3.2.1 Patient Characteristics

The 1036 NTE patients had a mean age of 74.9 years, and more than 30.4% (*n* = 315) were aged ≥ 80 years. Among these patients, 65.9% (*n* = 548) were considered frail, 33.6% had an ECOG‐PS ≥ 2 and almost half (47.1%) had comorbidities (Table 1). Severe renal impairment was observed in 13% of patients (*n* = 118) at treatment initiation and was more frequently associated with an ECOG‐PS ≥ 2 (47.7% vs. 31.7%; *p* < 0.01), ISS Stage III (83.3% vs. 41.3%; *p* < 0.0001) and comorbidities (65.3% vs. 44.8%; *p* < 0.0001) (Table S6).

Frailty was a key criterion for therapeutic decisions in NTE patients. In line with the frailty definition, 50.7% (278/548) of frail patients were aged ≥ 80 years, 54.6% (249/456) had a deteriorated ECOG‐PS and 56.2% (308/548) had relevant comorbidities, including 13.0% (71/548) of patients with a CCI ≥ 3.

A subgroup of 126 younger patients (age < 65 years) was considered ineligible for transplantation (23.8% of all patients with NDMM were aged < 65 years) although their age range was theoretically compatible with high‐dose treatment. They had a worse ECOG‐PS (≥ 2; 35.7% vs. 14.4% for patients aged < 65 years who received ASCT; *p* < 0.0001), more severe ISS Stage (Stage III in 47.3% of patients vs. 30.7%; *p* = 0.02), more comorbidities (≥ 1 in 38.9% of patients vs. 21.9%; *p* < 0.0001) and had more frequently severe renal failure (15.1% of patients vs. 3.7%, *p* < 0.0001) (Table S7).

Regarding cytogenetic testing, approximately half (56.4%) of the patients were tested in regular practice. Testing was carried out in the youngest patients (mean age 73.3 years) vs. the oldest patients (mean age 75.9 years) (*p* < 0.0001). Clinical practice appeared to vary by hospital for NTE patients, with more tests conducted in university hospitals than in general hospitals and private clinics (*p* < 0.0001) (Table S8).

#### 3.2.2 Therapeutic Management

During the study period, NTE patients mostly received one of the three main combinations, including two triplet regimens, VRd (28.4% of patients, *n* = 294) and VMp (21.9% of patients; *n* = 227) and a doublet combination of Rd. (21.1%, *n* = 219) (Table 2). While the total number of patients treated with each alternative was similar, their trajectories over the 2017–2020 period diverged. VMp use decreased from 36.9% to 6.5% of patients, VRd use increased from 10.6% to 48.5% and Rd. use remained more or less constant, at around 20% (Figure 1B). R‐based regimens were increasingly used over time, from 29.4% receiving R in upfront therapy in 2017 to 73.3% in 2020. VRd became the first therapeutic option for NTE patients as part of upfront therapy (half of all patients in 2020), just before the market introduction of Dara. Other bortezomib‐based regimens, including Vd and VCd, were steady, at around 12% and 7% of patients over the period, respectively.

Doublet therapy (Rd in 34.9% and Vd in 14.6%) was favoured for older patients (age ≥ 80 years) compared with the VRd option (4.4%). Similarly, frail patients were more likely to receive PI‐based (48.7% vs. 28.2%) or IMiD‐based (27.7% vs. 14.4%) regimens than IMiD + PI‐based treatments (19.0% vs. 55.6%) (*p* < 0.0001) (Table S9). PI‐based therapies comprised VMp in 133 frail patients (24.3%), VCd in 75% (13.7%) and Vd in 56 (10.2%).

Vd and VCd therapies were prioritised for patients with severe renal failure, with 39.0% vs. 8.4% receiving VCd and 20.3% vs. 5.6% receiving Vd, whereas 17.8% vs. 29.7%, and 9.3% vs. 23.5% received VRd and Rd., respectively. Vd and VCd were used in patients with a median age of 82 and 75.9 years, respectively, with ECOG‐PS ≥ 2 in 51.9% and 37.2%, and comorbidities in 50.7% and 58.6%, respectively.

HR NTE patients received similar treatment to non‐HR NTE patients (VRd 29.9%, VMp 26.0% and Rd. 20.8%) (Table S10). NTE patients aged < 65 years mainly received IMiD‐based triple therapy, firstly VRd (46.8% of patients vs. 25.8% for other NTE patients), followed by VTd (19.0% vs. 1.5%). Dual therapies were not used in this group of patients (apart from one patient who had Rd. and three who had Vd).

### 3.3 Survival Results

**FIGURE 2:**
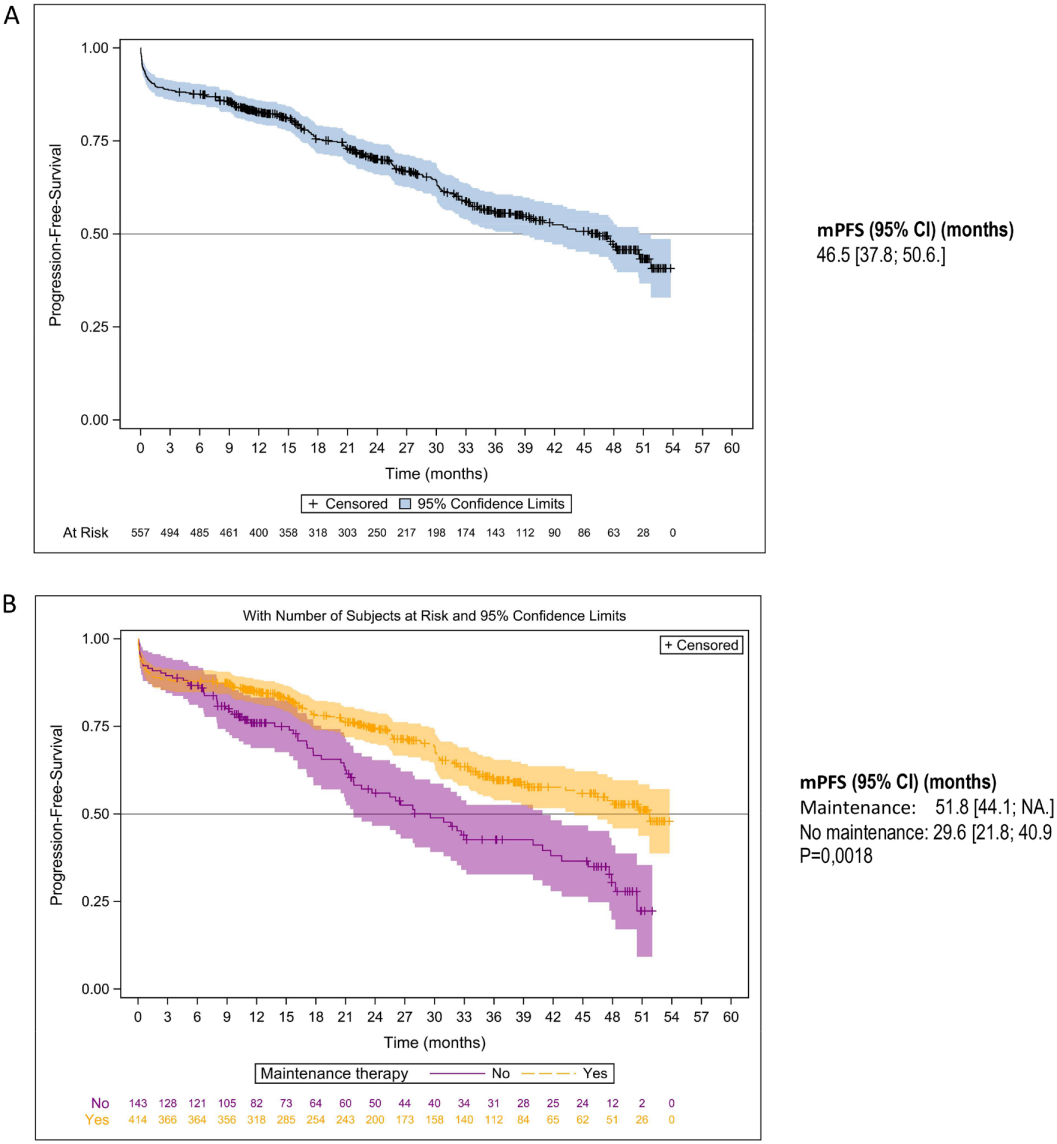
PFS distributions with 95% confidence intervals in (A) NDMM patients who received ASCT; (B) ASCT patients with maintenance and no maintenance therapy after ASCT.

**FIGURE 3:**
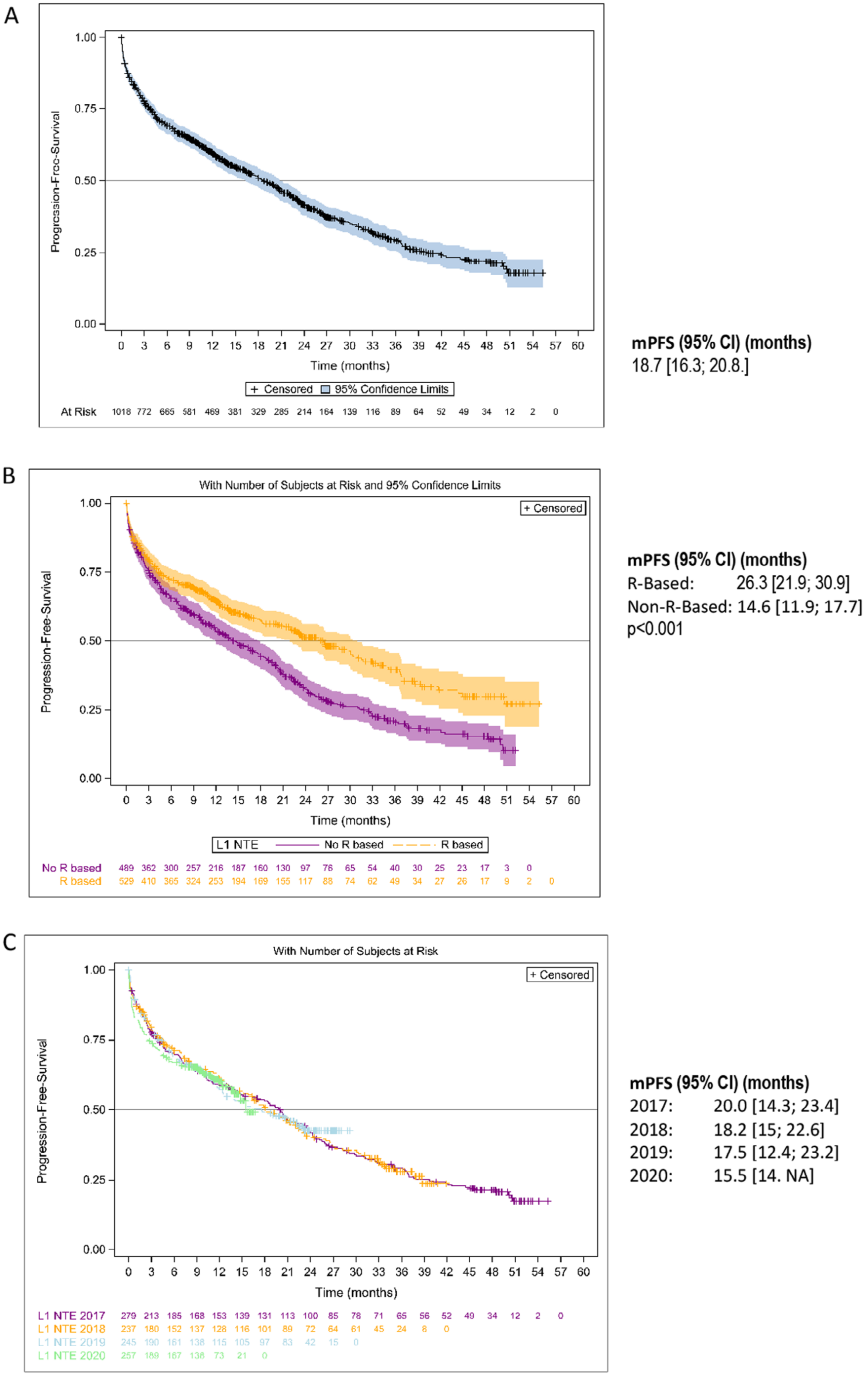
PFS distributions with 95% confidence intervals in (A) NDMM NTE patients; (B) NTE patients who received R‐ and non‐R‐based treatment; (C) NTE patients who initiated upfront therapy in 2017, 2018, 2019 and 2020.

The Kaplan–Meier estimates of PFS are shown in Figures 2 and 3. Median PFS (mPFS) was 25.8 months (95% CI: 23.5–29.1) in the overall NDMM population, 46.5 months (95% CI: 37.8–50.6) in patients who received ASCT, and 18.7 months (95% CI: 16.3–20.8) in NTE patients (Figures 2A and 3A). In the ASCT population, patients who received induction with VRd had an mPFS of 50.6 months (95% CI: 35.7– not achieved [NA]) and those who received VTd an mPFS of 45.5 months (95% CI: 34.2–50.5) (*p* = 0.47). Patients who received maintenance therapy after ASCT had an mPFS of 51.8 months (95% CI: 44.1–NA) and those without maintenance therapy an mPFS of 29.6 months (95% CI: 21.8–40.9) (*p* = 0.0018) (Figure 2B). In the NTE population, mPFS was 26.3 months (95% CI: 21.9–30.9) in patients who received an R‐based regimen compared with 14.6 months (95% CI: 11.9–17.7) in patients who had received a non‐R‐based regimen (*p* < 0.001) (Figure 3B). The year of enrolment in the cohort showed no impact on mPFS at the time of the analysis (Figure 3C). Non‐frail and frail NTE patients had an mPFS of 26.5 months (95% CI: 22.8–32.9) and 16.6 months (95% CI: 13.4–20.1), respectively.

The median TTNT was 32.6 months (95% CI: 29.1–34.7) in the overall population, including 52.1 months (95% CI: 48.4– NA) in patients who received ASCT and 20.4 months (95% CI: 18.3–22.8) in NTE patients (Table S11). In the NTE population, mTTNT was 30.3 months (95% CI: 25.8–37.7) in patients who received an R‐based regimen and 14.9 months (95% CI: 13.0–17.3) in patients who had received a non‐R‐based regimen (Table S12).

**FIGURE 4:**
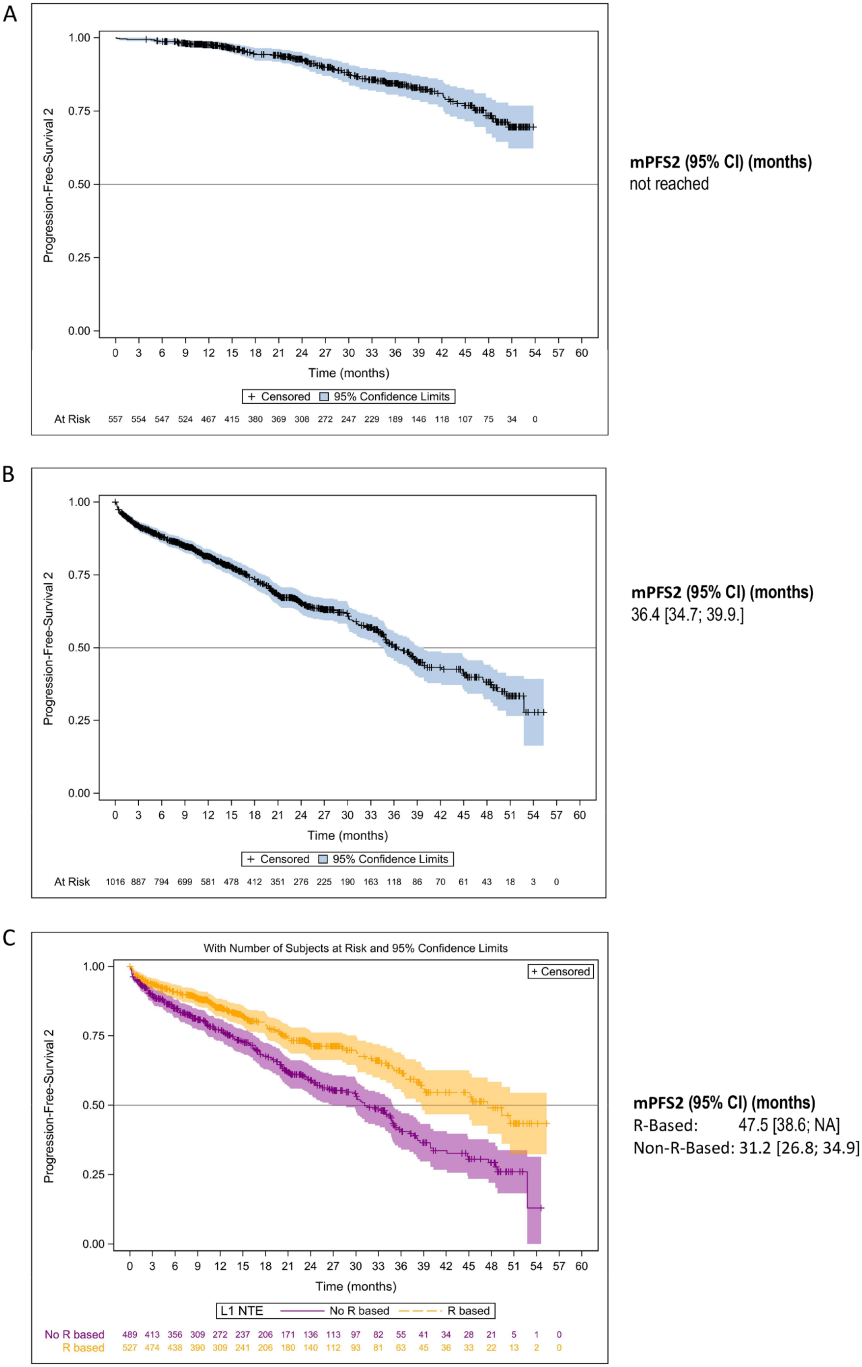
PFS2 distributions with 95% confidence intervals(A) in patients with NDMM who received ASCT; (B) in NTE patients with NDMM; (C) NTE patients who received R‐ and non‐R‐based treatment as first‐line therapy.

The median PFS2 was estimated at 50.6 months (95% CI: 47.6–NA) in all patients with NDMM and 36.4 months (95% CI: 34.7–39.9) in NTE patients; it was not reached in the ASCT group (Figure 4A,B). In the NTE population, mPFS2 was 47.5 months (95% CI: 38.6– NA) in patients who received an R‐based regimen compared with 31.2 months (95% CI: 26.8–34.9) in patients who had received a non‐R‐based regimen (Figure 4C).

In the ASCT population, the best response to first‐line treatment was≥very good PR (VGPR) in 84.8%, PR in 5.7%, and stable disease (SD) in 3.4% of patients with an available response assessment (*n* = 528). In the NTE population, the best response to first‐line treatment was≥VGPR in 53%, PR in 17% and SD in 6.2% of patients with an available response assessment (*n* = 917).

**FIGURE 5:**
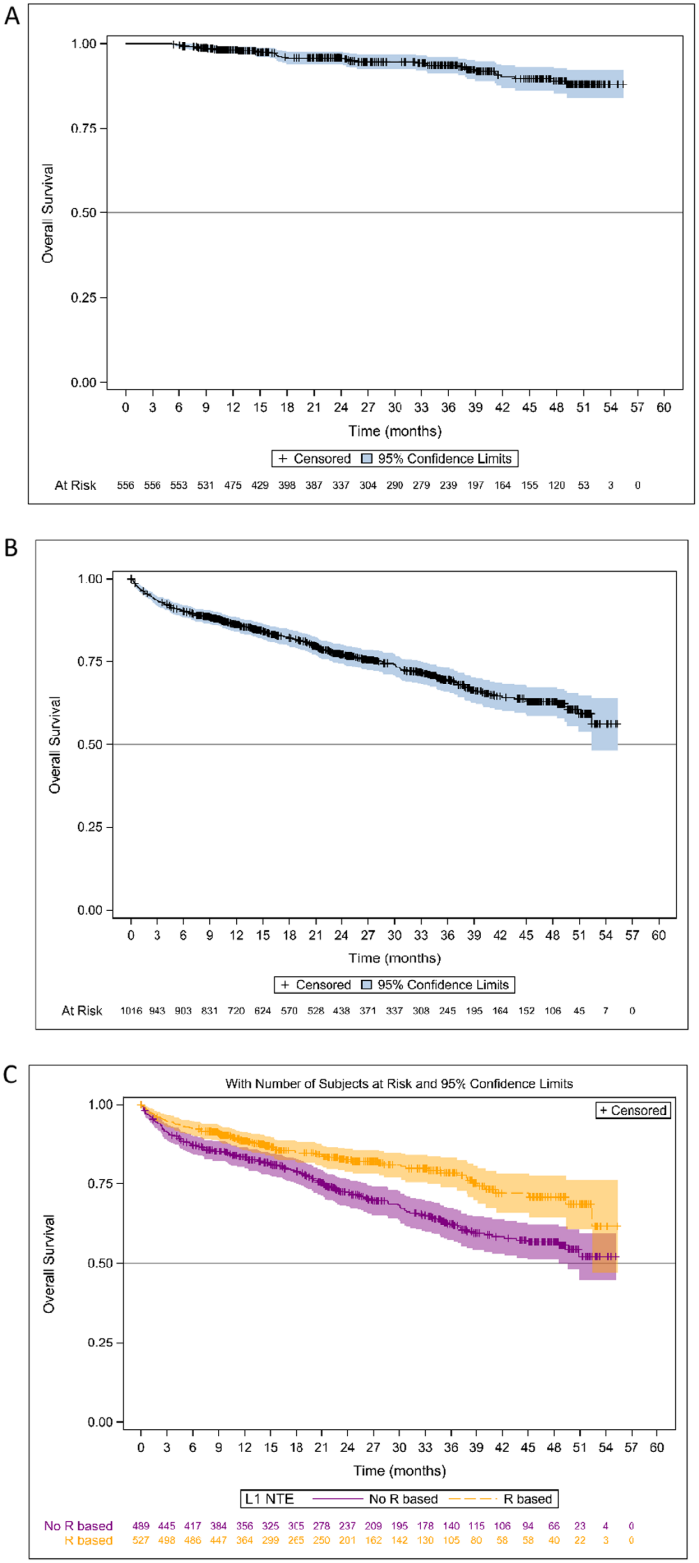
Overall survival (OS) distributions with 95% confidence intervals (A) in patients with NDMM who received ASCT; (B) in NTE patients with NDMM and (C) in NTE patients who received R‐ and non‐R‐based first‐line treatment.

At the time of the present analysis, the mOS had not yet been reached. The estimated OS rate was 90.4% (95% CI: 88.9–91.8) at 12 months, 83.9% (95% CI: 82–85.9) at 24 months, 78.5% (95% CI: 76.1–81) at 36 months and 72.9% (95% CI: 69.8–75.9) at 48 months for the overall NDMM population. The estimated OS for the patients who received ASCT and the NTE patients are presented in Figure 5A,B. The estimated 48‐month OS rates were 89% (95% CI: 84.5–92.2) for patients with ASCT and 63% (95% CI: 58.5–67.1) for NTE patients. In the ASCT patients, 48‐month OS rates were 88.2% (95% CI: 82.6–93.6) and 90.8% (95% CI: 84.9–96.8) in patients who received induction treatment with VTd and VRd, respectively. The 48‐month OS rates were 92.5% (95% CI: 88.5–96.4) and 79.3% (95% CI: 70.6–87.9) in patients with and without maintenance therapy after ASCT, respectively. In the NTE population, 48‐month OS rates were 70.8% (95% CI: 64.2–77.4) in patients who received an R‐based regimen and 56.7% (95% CI: 51.1–62.3) in patients who had received a non‐R‐based regimen (Figure 5C).

### 3.4 Safety

At the time of the analysis, 288/561 (51.3%) patients who received ASCT had permanently discontinued first‐line treatment, including 165 (29.1%) discontinuations as per protocol, 68 (12.1%) due to progression and 38 (6.8%) for adverse events (AEs). Reported AEs leading to discontinuation occurred at a median of 14.4 months after the start of first‐line treatment while patients were on maintenance therapy with R (13/15 treatment‐related AEs).

For the NTE group, 710/1036 (68.5%) patients discontinued treatment, 193 (18.6%) as per protocol, 264 (25.5%) due to progression and 150 (14.5%) due to AEs. AEs leading to discontinuation occurred at a median of 4.3 months after treatment initiation and were mainly reported with the most used agents (R, bortezomib or melphalan). The most frequently reported AEs leading to discontinuation were neuropathy (*n* = 14; 10.1% of the 139 patients reporting an AE leading to discontinuation), digestive disorders (*n* = 13; 9.4%), toxicoderma (*n* = 12; 8.6%), deterioration of general condition (*n* = 12; 8.6%), cardiovascular (*n* = 10; 7.2%) and acute renal failure (*n* = 10; 7.2%). The discontinuation rates due to AE were 14.5% in NTE patients who received non‐R‐based treatment (*n* = 73/497 patients) and R‐based treatment (*n* = 78/539).

## 4 Discussion

With nearly 1600 NDMM patients included in the study over a recent 4‐year period (from 2017 to 2020), whatever the initial therapy, EMMY is a unique longitudinal real‐life cohort in Europe and worldwide. It provides valuable insights into the day‐to‐day practice of clinicians and how this is evolving with the introduction of new therapeutic advances, both for patients receiving ASCT and for NTE patients. EMMY meets the challenge of recruiting a broad panel of patients into a cohort with fewer restrictions than those associated with a clinical trial and comprises a large proportion of elderly, frail or comorbid patients. By the end of 2020, over 1000 NTE patients and 500 patients receiving ASCT had been included in EMMY. Recruitment has been regular over each year, which is particularly meaningful for describing various subgroups of interest.

The study shows that therapeutic management in first‐line was relatively standard and based on a core of selected strategies, after deciding whether or not the patient was eligible for a transplantation. For patients receiving ASCT, the induction phase was based on a triple combination of bortezomib, an IMiD and d in almost 85% of cases. The estimated mPFS of 46.5 months is consistent with that of clinical trials (47.2 months in a long‐term analysis for IFM2009) [37]. The response rate was also quite high and confirmed efficacy of the ASCT strategy in real‐life. In 2017, thalidomide was still widely used, whereas in 2018, R became the reference IMiD following the results of the IFM2009 study [10], mainly for safety reasons. Post‐transplantation, the use of R as maintenance therapy became the standard (over 80% of patients as of 2018) in accordance with the ESMO (European Society for Medical Oncology) recommendations (2; 5) and clinical evidence [38]. EMMY confirms, in real‐life, the clinical benefit of maintenance with R in patients who received VTd and VRd induction therapies.

NTE patients mostly received an R‐ or V‐based combination including Rd. (28%), VRd (21%), VMp (22%) and to a lesser extent VCd (12%) or Vd (7%) regimens. Similar to the ASCT group, R‐based first‐line treatments were increasingly used in NTE patients from 2018 (over 70% in 2020) while V‐based combinations, particularly VMp, significantly declined. With the SWOG S0777 clinical results [12], VRd became the frontline treatment of choice while Dara has not yet been approved as a first‐line therapy in Europe. Although the analysis was not adjusted for patient characteristics, the benefit of first‐line R was significant, with an improvement in mPFS of 12 months (from 14.6 months to 26.3 months) between non‐R‐based and R‐based patients. The mPFS in R‐based patients is close to that of clinical trials for Rd. first‐line (29 months vs. 26 months in the FIRST trial) but remains lower than expected with VRd (41 months in long‐term analysis of SWOG S0777) [39]. This result has already been observed in the FLATIRON MM registry [30] with a mPFS of 26.5 months in real‐life patients receiving VRd in the US suggesting that VRd is mainly of interest for fit NTE patients. A specific EMMY analysis of VRd vs. Rd. in NDMM NTE patients has shown a real benefit of VRd in patients under 75 years of age (29.4 months for Rd. and 34.1 months for VRd) [31].

Besides these major trends in managing patients with NDMM, specific therapeutic subgroups have also been identified. Thus, in line with current recommendations [5], almost half of patients aged between 65 and 69 (46%) years and a further 10% of those aged ≥ 70 years, received ASCT. While no clinical trials have demonstrated efficacy and tolerability of ASCT in those aged ≥ 65 years, EMMY is among the first retrospective studies to do so and has shown comparable efficacy as in younger patients [33]. A retrospective study on 53 patients ≥ 74 years provided a rationale for offering ASCT to elderly patients [40]. Conversely, a group of 126 young patients (aged < 65 years) did not receive ASCT. Some of these patients were not able to be treated during the COVID‐19 pandemic years of 2019 and 2020, but most were medically fragile and did not meet the criteria for high‐dose therapy followed by transplantation, demonstrating the range of treatment decisions that clinicians are faced with. In the ASCT group, the recourse to double transplantation remains limited (less than 10% of patients) and stable over time [41]. This option was mainly reserved for patients with HR MM in EMMY, for whom the benefit was more clearly demonstrated [42].

Over 300 EMMY patients were diagnosed at advanced age, resulting in 30% of NTE NDMM patients in the cohort being aged ≥ 80 years. These unique data for very elderly patients, poorly represented in clinical trials, confirmed that dual therapies were favoured as frontline in this population (Rd 35% or Vd 15%), with VRd almost completely excluded. Results are consistent with EMMY's previous findings for patients aged ≥ 75 years suggesting that VRd did not provide significant benefit vs. Rd. for those patients [31]. More generally, when age, unfavourable ECOG score and relevant comorbidities are all considered, over 500 NTE patients were identified as frail and required adaptive therapeutic care according to their health status [43, 44, 45]. For those patients, EMMY reported that VRd use remained limited with a notable preference for doublet R‐based therapies and to a larger extent to V‐based therapies, which remained of interest in specific situations such as severe renal failure.

In the real‐world setting, tests for cytogenetic abnormalities are not systematically carried out in NDMM patients. Results from EMMY were available for two‐thirds of patients, this being consistent with other real‐life cohorts in which between 60% and 69% of patients (CONNECT MM registry) [46] and 75% of patients (FLATIRON MM registry) [30] had cytogenetics performed. Tests were frequently carried out for patients eligible for ASCT, but only for half of NTE patients, with no significant increase over the years. Among NTE patients, cytogenetic testing was more likely to be carried out for younger patients for whom a longer‐term treatment strategy or enrolment in future clinical trials should be considered.

Five years after EMMY had started (median follow‐up of 24 months), the mOS has not yet been achieved for NDMM patients. In recent real‐life analyses, it has been estimated at over 60 months for patients diagnosed between 2013 and 2019 and around 64 months for the subgroup of patients diagnosed between 2017 and 2019 [47]. To date, the 48‐month OS of 73% in EMMY patients, 89% for ASCT patients and 63% for NTE patients, is consistent with other studies (10;12). There is now sufficient follow‐up to estimate mPFS2, particularly in NTE patients (36 months) which means that the benefit of ‘first‐line—first relapse’ treatment sequences can be assessed more accurately in real‐life.

The EMMY study has some limitations, particularly those inherent to real‐life studies based on patients' medical records. Data are collected based on availability, examinations carried out and investigations conducted by each centre. Over the years the records of patients participating in EMMY have increasingly been standardised, but differences may persist, particularly in the definition of outcomes and in the frequency of routine patients' visits to hospitals. Also, while EMMY has focused extensively on current practice and the benefits of treatments, safety data are more limited and the toxicity of treatments is not often described, except when associated with treatment discontinuation. The representativeness of patients in studies remains an important issue. To achieve these objectives, the identification of all eligible EMMY patients is thorough, and the process is monitored at the participating sites each year.

## 5 Conclusion

These results provide an overview of how NDMM patients are managed in the years prior to the emergence of Dara in first‐line, largely dominated by R‐ and in particular VR‐based treatments for eligible patients. Although the literature contains studies that focus on a particular therapeutic strategy such as VRd induction or initial intent of first‐line transplant (30; 43), there has been no description, to our knowledge, of the evolution of NDMM management over the years in recent similar studies.

The description of NDMM patients from the EMMY cohort provides extensive information and shows that first‐line treatment standards are continuously evolving as clinical evidence improves while also needing to be adapted to real‐life patients' clinical profiles and their ability to receive the most effective treatment for their disease.

## Author Contributions

**Laure Vincent:** formal analysis (supporting), investigation (equal), validation (equal), writing – original draft (lead). **Olivier Decaux:** conceptualization (lead), formal analysis (supporting), investigation (equal), methodology (equal), supervision (lead), writing – original draft (equal). **Aurore Perrot:** conceptualization (supporting), formal analysis (supporting), investigation (equal), supervision (supporting), writing – original draft (supporting). **Bruno Royer:** investigation (equal), writing – original draft (supporting). **Thomas Chalopin:** investigation (equal), validation (supporting), writing – original draft (supporting). **Arthur Bobin:** investigation (equal), writing – original draft (supporting). **Margaret Macro:** conceptualization (equal), formal analysis (supporting), investigation (equal), methodology (equal), supervision (equal), writing – original draft (supporting). **Denis Caillot:** investigation (equal), writing – original draft (supporting). **Lionel Karlin:** investigation (equal), writing – original draft (supporting). **Caroline Jacquet:** investigation (equal), writing – original draft (supporting). **Cécile Sonntag:** investigation (equal), writing – original draft (supporting). **Mohamad Mohty:** investigation (equal), writing – original draft (supporting). **Laurent Frenzel:** conceptualization (supporting), investigation (equal), writing – original draft (supporting). **Arnaud Jaccard:** investigation (equal), writing – original draft (supporting). **Salomon Manier:** investigation (equal), writing – original draft (supporting). **Laurence Sanhes:** investigation (equal), writing – original draft (supporting). **Driss Chaoui:** investigation (equal), writing – original draft (supporting). **Philippe Moreau:** investigation (equal), supervision (equal), writing – original draft (supporting). **Ronan Garlantézec:** conceptualization (equal), formal analysis (supporting), methodology (equal), writing – original draft (supporting). **Nathalie Texier:** conceptualization (equal), methodology (equal), supervision (equal), writing – original draft (equal). **Chanaz Louni:** conceptualization (equal), supervision (equal), writing – original draft (supporting). **Zakaria Maarouf:** conceptualization (equal), project administration (equal), supervision (equal), writing – original draft (equal). **Herve Avet Loiseau:** supervision (equal), writing – original draft (supporting). **Cyrille Hulin:** conceptualization (equal), formal analysis (equal), investigation (equal), methodology (equal), writing – original draft (equal). **Karim Belhadj Merzoug:** formal analysis (equal), investigation (equal), writing – original draft (lead).

## Ethics Statement

The EMMY study is classified as research not involving human subject as defined in article L. 1121–1 of the French Public Health Code. This retrospective and prospective personal data study has been submitted to the French Health Data (N° F20220518161845, https://www.health‐data‐hub.fr/projets/epidemiology‐therapeutic‐management‐multiple‐myeloma‐france‐emmy‐project) Epidemiology of the therapeutic management of multiple myeloma in France—EmmY Project | Health Data Hub (health‐data‐hub.fr) in accordance with local regulations. No ethical approval for this study was required.

## Consent

All patients were informed about the processing of their data using a written information letter prior to enrolment in the study and did not oppose the study and the data collection as per local regulations in France for research not involving human subject as defined in article L. 1121–1 of the French Public Health Code. Patients' choice is documented in investigators sites in accordance with good practice in epidemiology before being enrolled.

## Conflicts of Interest

L.V. received honoraria from Janssen, BMS, Takeda, Sanofi, Pfizer; O.D. received honoraria from Janssen, BMS, GSK, Sanofi, Takeda, Roche and Gilead; A.P. received honoraria from Abbvie, Amgen, BMS, GSK, Janssen, Sanofi and Takeda; M.M. received honoraria from Janssen, Sanofi, GSK and Takeda; L.K. received honoraria from Amgen, BMS, Janssen, Takeda, Sanofi, Abbvie, Stemline and GSK; C.S. participate as expert in board of experts for BMS, Amgen, Janssen, Takeda and Sanofi; M.M. received honoraria from Janssen, Sanofi, BMS, Amgen, Takeda, GSK, Pfizer, Novartis, Jazz Pharmaceuticals, Stemline and Astellas; L.F. is a consultant for Pfizer, Roche, CSL Behring, Sobi, BioMarin Pharmaceutical, A.J. received honoraria from Abbvie, Pfizer and Janssen; S.M. is a consultant for AbbVie, Adaptative Biotechnology, Amgen, BMS, GSK, Janssen, Regeneron, Roche, Sanofi, Takeda; P.M. is a consultant and received honoraria from Janssen, BMS, Amgen, Abbvie, Sanofi and Takeda; L.C. et ZM are employees of IFM; C.H. received honoraria from Janssen, BMS, GSK, Takeda, Sanofi and Amgen; K.B.M. is a consultant and received honoraria for BMS, Janssen, Sanofi, Amgen and Abbvie. The other authors declare that they have no competing interests.

## Supporting information


Data S1.

## Data Availability

The datasets generated during and/or analysed during the current study are available from the corresponding author on reasonable request.
