# Supplementary material for: Real‐World Outcomes of Newly Diagnosed Multiple Myeloma Patients Treated Before the Era of Anti‐CD38 Antibodies: The EMMY Cohort From 2017 to 2020
Source: Cancer Med. 2025 Mar 14;14(6):e70619. doi: 10.1002/cam4.70619 (PMC11909396; doi:10.1002/cam4.70619)
Supplement: Supplementary file 1 — Data S1. [file CAM4-14-e70619-s001.docx]

# Supplemental Data -

# ASCT Patients

### Supplemental Table 1: Severe renal impairment at start of initial treatment in ASCT patients (N=561)

|  | | **ASCT – Renal impairment** | | **p** |
| --- | --- | --- | --- | --- |
|  | | **No* (N=543)** | **Yes** (N=18)** |  |
| **Age (years)** | Median | 60.7 | 57.7 | 0.16 |
|  | IQR | 55 – 65.5 | 51.1 – 64.2 |  |
|  | | | | |
| **Gender, n (%)** | Male | 328 (60.4%) | 10 (55.6%) | 0.68 |
|  | Female | 215 (39.6%) | 8 (44.4%) |  |
|  | | | | |
| **ECOG-PS, n (%)** |  | 426 | 10 | 0.12 |
|  | 0 - 1 | 371 (87.1%) | 7 (70.0%) |  |
|  | ≥ 2 | 55 (12.9%) | 3 (30.0%) |  |
|  | | | | |
| **ISS, n (%)** |  | 347 | 13 | <0.001 |
|  | Stage I | 127 (36.6%) | 0 (0.0%) |  |
|  | Stage II | 120 (34.6%) | 2 (15.4%) |  |
|  | Stage III | 100 (28.8%) | 11 (84.6%) |  |
|  | | | | |
| **Cytogenetic test, n (%)** |  | 459 | 14 | 0.71 |
|  | Yes | 348 (75.8%) | 10 (71.4%) |  |
|  | | | | |
| **If yes. high cytogenetic risk, n (%)** | Yes | 69 (19.8%) | 1 (10.0%) | 0.44 |
|  | | | | |
| **Comorbidities, n (%)** | 0 | 417 (76.8%) | 12 (66.7%) | 0.06 |
|  | 1 | 102 (18.8%) | 3 (16.7%) |  |
|  | >=2 | 24 (4.4%) | 3 (16.7%) |  |
|  | | | | |
| *Glomerular filtration rate (GFR) ≥30 mL/min or missing; ** GFR<30 mL/min | | | | |

### Supplemental Table 2: Cytogenetic test at start of initial treatment in ASCT patients (N=561)

|  | | **ASCT – Cytogenetic test** | | | **p** |
| --- | --- | --- | --- | --- | --- |
|  | | **No (N=115)** | **Yes (N=358)** | **Missing data (N=88)** |  |
| **Age (years)** | Median | 59.1 | 60.7 | 62.5 | 0.14 |
|  | IQR | 52 – 65.1 | 55.6 – 65.2 | 55.4 – 67.4 |  |
|  | | | | | |
| **Gender, n (%)** | Male | 71 (61.7%) | 220 (61.5%) | 47 (53.4%) | 0.36 |
|  | Female | 44 (38.3%) | 138 (38.5%) | 41 (46.6%) |  |
|  | | | | | |
| **ECOG-PS, n (%)** |  | 95 | 287 | 54 | 0.10 |
|  | 0 - 1 | 82 (86.3%) | 254 (88.5%) | 42 (77.8%) |  |
|  | ≥2 | 13 (13.7%) | 33 (11.5%) | 12 (22.2%) |  |
|  | | | | | |
| **ISS, n (%)** |  | 71 | 239 | 50 | 0.77 |
|  | Stage I | 26 (36.6%) | 87 (36.4%) | 14 (28.0%) |  |
|  | Stage II | 25 (35.2%) | 81 (33.9%) | 16 (32.0%) |  |
|  | Stage III | 20 (28.2%) | 71 (29.7%) | 20 (40.0%) |  |
|  | | | | | |
| **High cytogenetic risk, n (%)** | Yes | 0 (0.0%) | 70 (19.6%) | 0 (0.0%) | <0.0001 |
|  | | | | | |
| **Comorbidities, n (%)** | 0 | 93 (80.9%) | 270 (75.4%) | 66 (75.0%) | 0.49 |
|  | 1 | 19 (16.5%) | 67 (18.7%) | 19 (21.6%) |  |
|  | >=2 | 3 (2.6%) | 21 (5.9%) | 3 (3.4%) |  |
|  | | | | | |
| **Year of L1 initiation, n (%)** | 2017 | 35 (30.4%) | 139 (38.8%) | 24 (27.3%) | 0.36 |
|  | 2018 | 30 (26.1%) | 86 (24.0%) | 23 (26.1%) |  |
|  | 2019 | 24 (20.9%) | 57 (15.9%) | 20 (22.7%) |  |
|  | 2020 | 26 (22.6%) | 76 (21.2%) | 21 (23.9%) |  |
|  | | | | | |
| **Type of center, n (%)** | CHU | 67 (58.3%) | 227 (63.4%) | 51 (58.0%) | 0.17 |
|  | CH-CHG | 39 (33.9%) | 96 (26.8%) | 33 (37.5%) |  |
|  | Private clinic | 9 (7.8%) | 35 (9.8%) | 4 (4.5%) |  |
|  | | | | | |

### Supplemental Table 3: Description of treatment according to high risk (HR) and non-high risk status in ASCT patients (N=561)

|  | | **ASCT** | | **p** | |
| --- | --- | --- | --- | --- | --- |
|  | | **non HR* (N=491)** | **HR (N=70)** |  | |
| **Combinations. n (%)** | bortezomib; lenalidomide +/- corticoids // melphalan +/- corticoids | 276 (56.2%) | 33 (47.1%) |  | |
|  | bortezomib; thalidomide +/- corticoids // melphalan +/- corticoids | 140 (28.5%) | 23 (32.9%) |  | |
|  | bortezomib; cyclophosphamide +/- corticoids // melphalan +/- corticoids | 22 (4.5%) | 6 (8.6%) |  | |
|  | bortezomib; cyclophosphamide; lenalidomide +/- corticoids // melphalan +/- corticoids | 10 (2.0%) | 1 (1.4%) |  | |
|  | bortezomib; daratumumab; lenalidomide +/- corticoids // melphalan +/- corticoids | 3 (0.6%) | 4 (5.7%) |  | |
|  | bortezomib +/- corticoids // melphalan +/- corticoids | 5 (1.0%) | 0 (0.0%) |  | |
|  | bortezomib; lenalidomide +/- corticoids // cyclophosphamide +/- corticoids | 4 (0.8%) | 0 (0.0%) |  | |
|  | bortezomib; lenalidomide +/- corticoids // non connu | 4 (0.8%) | 0 (0.0%) |  | |
|  | bortezomib; cyclophosphamide; thalidomide +/- corticoids // melphalan +/- corticoids | 2 (0.4%) | 1 (1.4%) |  | |
|  | bortezomib; lenalidomide +/- corticoids (VRD) | 3 (0.6%) | 0 (0.0%) |  | |
|  | bortezomib; daratumumab; thalidomide +/- corticoids // melphalan +/- corticoids | 2 (0.4%) | 0 (0.0%) |  | |
|  | bortezomib; lenalidomide +/- corticoids // cyclophosphamide; melphalan +/- corticoids | 2 (0.4%) | 0 (0.0%) |  | |
|  | bortezomib; lenalidomide; thalidomide +/- corticoids // melphalan +/- corticoids | 2 (0.4%) | 0 (0.0%) |  | |
|  | bortezomib; thalidomide +/- corticoids // cyclophosphamide +/- corticoids | 2 (0.4%) | 0 (0.0%) |  | |
|  | Other | 14 (2.9%) | 2 (2.9%) |  | |
|  | | | | |  |
| **Proteasome inhibitor. n (%)** | | **488 (99.4%)** | **70 (100.0%)** | **0.51** | |
|  | Bortezomib | 488 (99.4%) | 70 (100.0%) |  | |
| **IMID. n (%)** |  | 460 (93.7%) | 64 (91.4%) | 0.48 | |
|  | Thalidomide | 153 (31.2%) | 26 (37.1%) |  | |
|  | Lenalidomide | 308 (62.7%) | 38 (54.3%) |  | |
|  | Pomalidomide | 1 (0.2%) | 0 (0.0%) |  | |
| **Anti-CD38. n (%)** | | **7 (1.4%)** | **4 (5.7%)** | **0.02** | |
|  | Daratumumab | 7 (1.4%) | 4 (5.7%) |  | |
| **Alkylant. n (%)** |  | **480 (97.8%)** | **69 (98.6%)** |  | |
|  | Melphalan | 473 (96.3%) | 69 (98.6%) |  | |
|  | Cyclophosphamide | 48 (9.8%) | 9 (12.9%) |  | |
|  | Bendamustine | 1 (0.2%) | 0 (0.0%) |  | |
|  | Other cytotoxics | 2 (0.4%) | 0 (0.0%) |  | |
|  | | | | |  |
| **Category. n (%)** | Anti-CD38 +/- others | 7 (1.4%) | 4 (5.7%) | 0.07 | |
|  | IMID + PI +/- others | 451 (91.9%) | 60 (85.7%) |  | |
|  | IMID +/- others | 3 (0.6%) | 0 (0.0%) |  | |
|  | PI +/- others | 30 (6.1%) | 6 (8.6%) |  | |
|  | Anti-CD38 +/- others | 0 (0.0%) | 0 (0.0%) |  | |
|  | | | | |  |

**includes non-HR patients and patients with an unknown cytogenetic feature (not done)*

### Supplemental Table 4: Single versus double transplantation at the start of initial treatment in ASCT patients (N=561)

|  | | **ASCT** | | **p** |
| --- | --- | --- | --- | --- |
|  | | **Single (N=508)** | **Double (N=53)** |  |
| **Age (years)** | Median | 60.7 | 58.9 | 0.54 |
|  | IQR | 55 – 65.5 | 54.6 – 65.1 |  |
|  | | | |  |
| **Gender. n (%)** | Male | 305 (60.0%) | 33 (62.3%) | 0.75 |
|  | Female | 203 (40.0%) | 20 (37.7%) |  |
|  |  |  |  |  |
| **ECOG-PS. n (%)** |  | 395 | 41 | 0.83 |
|  | 0 - 1 | 342 (86.6%) | 36 (87.8%) |  |
|  | ≥2 | 53 (13.4%) | 5 (12.2%) |  |
|  | | | |  |
| **ISS. n (%)** |  | 323 | 37 | 0.30 |
|  | Stage I | 118 (36.5%) | 9 (24.3%) |  |
|  | Stage II | 110 (34.1%) | 12 (32.4%) |  |
|  | Stage III | 95 (29.4%) | 16 (43.2%) |  |
|  | | | |  |
| **Cytogenetic test. n (%)** |  | 425 | 48 | 0.04 |
|  | Yes | 316 (74.4%) | 42 (87.5%) |  |
|  | | | |  |
| **If yes. high cytogenetic risk. n (%)** | Yes | 54 (17.1%) | 16 (38.1%) | <0.01 |
|  | | | |  |
| **Comorbidities. n (%)** | 0 | 389 (76.6%) | 40 (75.5%) | 0.87 |
|  | 1 | 94 (18.5%) | 11 (20.8%) |  |
|  | >=2 | 25 (4.9%) | 2 (3.8%) |  |
|  |  |  |  |  |
| **Year of L1 initiation. n (%)** | 2017 | 184 (36.2%) | 14 (26.4%) | 0.13 |
|  | 2018 | 119 (23.4%) | 20 (37.7%) |  |
|  | 2019 | 93 (18.3%) | 8 (15.1%) |  |
|  | 2020 | 112 (22.0%) | 11 (20.8%) |  |
|  | | | |  |

### Supplemental Table 5: Description of treatment according to double transplantation at start of initial treatment in ASCT patients (N=561)

|  | | **ASCT** | | **p** |
| --- | --- | --- | --- | --- |
|  |  | **Single (N=508)** | **Double (N=53)** |  |
| **Combinations. n (%)** | bortezomib; lenalidomide +/- corticoids // melphalan | 275 (54.1%) | 34 (64.2%) |  |
|  | bortezomib; thalidomide +/- corticoids // melphalan | 155 (30.5%) | 8 (15.1%) |  |
|  | bortezomib; cyclophosphamide +/- corticoids // melphalan | 27 (5.3%) | 1 (1.9%) |  |
|  | bortezomib; cyclophosphamide; lenalidomide +/- corticoids // melphalan | 8 (1.6%) | 3 (5.7%) |  |
|  | bortezomib; daratumumab; lenalidomide +/- corticoids // melphalan | 3 (0.6%) | 4 (7.5%) |  |
|  | bortezomib +/- corticoids // melphalan | 5 (1.0%) | 0 (0.0%) |  |
|  | bortezomib; lenalidomide +/- corticoids // cyclophosphamide | 3 (0.6%) | 1 (1.9%) |  |
|  | bortezomib; lenalidomide +/- corticoids // unknown | 4 (0.8%) | 0 (0.0%) |  |
|  | bortezomib; cyclophosphamide; thalidomide +/- corticoids // melphalan | 3 (0.6%) | 0 (0.0%) |  |
|  | bortezomib; lenalidomide +/- corticoids | 3 (0.6%) | 0 (0.0%) |  |
|  | bortezomib; daratumumab; thalidomide +/- corticoids // melphalan | 2 (0.4%) | 0 (0.0%) |  |
|  | bortezomib; lenalidomide +/- corticoids // cyclophosphamide; melphalan | 2 (0.4%) | 0 (0.0%) |  |
|  | bortezomib; lenalidomide; thalidomide +/- corticoids // melphalan | 2 (0.4%) | 0 (0.0%) |  |
|  | bortezomib; thalidomide +/- corticoids // cyclophosphamide | 2 (0.4%) | 0 (0.0%) |  |
|  | Other | 14 (1.4%) | 2 (3.8%) |  |
|  | | | |  |
| **Proteasome inhibitor. n (%)** |  | **505 (99.4%)** | **53 (100.0%)** | 0.57 |
|  | Bortezomib | 505 (99.4%) | 53 (100.0%) |  |
| **IMID. n (%)** |  | **473 (93.1%)** | **51 (96.2%)** | 0.38 |
|  | Thalidomide | 170 (33.5%) | 9 (17.0%) |  |
|  | Lenalidomide | 304 (59.8%) | 42 (79.2%) |  |
|  | Pomalidomide | 1 (0.2%) | 0 (0.0%) |  |
| **Anti-CD38. n (%)** |  | **7 (1.4%)** | **4 (7.5%)** | <0.01 |
|  | Daratumumab | 7 (1.4%) | 4 (7.5%) |  |
| **Alkylant. n (%)** |  | **496 (97.6%)** | **53 (100.0%)** |  |
|  | Melphalan | 490 (96.5%) | 52 (98.1%) |  |
|  | Cyclophosphamide | 50 (9.8%) | 7 (13.2%) |  |
|  | Bendamustine | 1 (0.2%) | 0 (0.0%) |  |
|  | | | |  |
| **Category. n (%)** | Anti-CD38 +/- others | 7 (1.4%) | 4 (7.5%) | 0.02 |
|  | IMID + PI +/- others | 464 (91.3%) | 47 (88.7%) |  |
|  | IMID +/- others | 3 (0.6%) | 0 (0.0%) |  |
|  | PI +/- others | 34 (6.7%) | 2 (3.8%) |  |
|  | | | |  |

# NTE Patients

### Supplemental Table 6: Severe renal impairment at start of initial treatment in NTE patients (N=1036)

|  | | **NTE – Renal impairment** | | **p** |
| --- | --- | --- | --- | --- |
|  | | **No (N=918)** | **Yes (N=118)** |  |
| **Age (years)** | Median | 74.7 | 75.7 | 0.31 |
|  | IQR | 69.6 – 81.3 | 69.2 – 82.4 |  |
|  | | | | |
| **Gender. n (%)** | Male | 458 (49.9%) | 68 (57.6%) | 0.11 |
|  | Female | 460 (50.1%) | 50 (42.4%) |  |
|  | | | | |
| **ECOG-PS. n (%)** |  | 652 | 88 | <0.01 |
|  | 0 - 1 | 445 (68.3%) | 46 (52.3%) |  |
|  | ≥2 | 207 (31.7%) | 42 (47.7%) |  |
|  | | | | |
| **ISS. n (%)** |  | 501 | 54 |  |
|  | Stage I | 135 (26.9%) | 4 (7.4%) | <0.0001 |
|  | Stage II | 159 (31.7%) | 5 (9.3%) |  |
|  | Stage III | 207 (41.3%) | 45 (83.3%) |  |
|  | | | | |
| **Cytogenetic test. n (%)** |  | 746 | 84 |  |
|  | Yes | 428 (57.4%) | 40 (47.6%) | 0.09 |
|  | | | | |
| **If yes. high cytogenetic risk. n (%)** | Yes | 70 (16.4%) | 7 (17.5%) | 0.85 |
|  | | | | |
| **Comorbidities. n (%)** | 0 | 507 (55.2%) | 41 (34.7%) | <0.0001 |
|  | 1 | 245 (26.7%) | 34 (28.8%) |  |
|  | ≥2 | 166 (18.1%) | 43 (36.4%) |  |
|  | | | | |

### Supplemental Table 7: Patient demographics at baseline and disease characteristics in patients < 65 years

|  | | **< 65 years** | | **p** |
| --- | --- | --- | --- | --- |
|  | | **NTE (N=126)** | **ASCT (N=403)** |  |
| **Age (years)** | Median | 59.6 | 57.7 | <0.01 |
|  | IQR | 55.8 – 62.6 | 52.1 – 61.9 |  |
|  | | | | |
| **Gender. n (%)** | Male | 77 (61.1%) | 250 (62.0%) | 0.85 |
|  | Female | 49 (38.9%) | 153 (38.0%) |  |
|  | | | | |
| **ECOG-PS. n (%)** |  | 84 | 312 | <0.0001 |
|  | 0 - 1 | 54 (64.3%) | 267 (85.6%) |  |
|  | ≥2 | 30 (35.7%) | 45 (14.4%) |  |
|  | | | | |
| **ISS. n (%)** |  | 74 | 261 |  |
|  | Stage I | 22 (29.7%) | 97 (37.2%) | 0.02 |
|  | Stage II | 17 (23.0%) | 84 (32.2%) |  |
|  | Stage III | 35 (47.3%) | 80 (30.7%) |  |
|  | | | | |
| **Cytogenetic test. n (%)** |  | 106 | 347 | 0.12 |
|  | Yes | 72 (67.9%) | 262 (75.5%) |  |
|  | | | | |
| **If yes. high cytogenetic risk. n (%)** | Yes | 10 (13.9%) | 56 (21.4%) | 0.16 |
|  | | | | |
| **Comorbidities. n (%)** | 0 | 77 (61.1%) | 315 (78.2%) | <0.0001 |
|  | 1 | 32 (25.4%) | 70 (17.4%) |  |
|  | >=2 | 17 (13.5%) | 18 (4.5%) |  |
|  |  |  |  |  |
| **Renal clearance <30 ml/min. n (%)** | Yes | 19 (15.1%) | 15 (3.7%) | <0.0001 |
|  | | | | |

### Supplemental Table 8: Cytogenetic test at start of initial treatment in NTE patients (N=1036)

|  | | **NTE – Cytogenetic test** | | | **p** |
| --- | --- | --- | --- | --- | --- |
|  | | **No (N=362)** | **Yes (N=468)** | **Missing data (N=206)** |  |
| **Age (years)** | Median | 75.9 | 73.3 | 76.1 | <0.0001 |
|  | IQR | 70.4 – 82.7 | 68.3 – 79.6 | 70.9 – 82.4 |  |
|  | | | | | |
| **Gender. n (%)** | Male | 175 (48.3%) | 237 (50.6%) | 114 (55.3%) | 0.28 |
|  | Female | 187 (51.7%) | 231 (49.4%) | 92 (44.7%) |  |
|  | | | | | |
| **ECOG-PS. n (%)** |  | 269 | 334 | 137 | 0.39 |
|  | 0 - 1 | 171 (63.6%) | 230 (68.9%) | 90 (65.7%) |  |
|  | ≥2 | 98 (36.4%) | 104 (31.1%) | 47 (34.3%) |  |
|  | | | | | |
| **ISS. n (%)** |  | 179 | 281 | 95 | 0.69 |
|  | Stade I | 45 (25.1%) | 75 (26.7%) | 19 (20.0%) |  |
|  | Stade II | 56 (31.3%) | 81 (28.8%) | 27 (28.4%) |  |
|  | Stade III | 78 (43.6%) | 125 (44.5%) | 49 (51.6%) |  |
|  | | | | | |
|  | | | | | |
| **High cytogenetic risk. n (%)** | Yes | 0 (0.0%) | 77 (16.5%) | 0 (0.0%) | <0.0001 |
|  | | | | | |
| **Comorbidities. n (%)** | 0 | 193 (53.3%) | 245 (52.4%) | 110 (53.4%) | 0.68 |
|  | 1 | 94 (26.0%) | 135 (28.8%) | 50 (24.3%) |  |
|  | ≥2 | 75 (20.7%) | 88 (18.8%) | 46 (22.3%) |  |
|  |  |  |  |  |  |
| **Type of center. n (%)** | CHU | 158 (43.6%) | 293 (62.6%) | 118 (57.3%) | <0.0001 |
|  | CH-CHG | 161 (44.5%) | 139 (29.7%) | 77 (37.4%) |  |
|  | Private clinic | 43 (11.9%) | 36 (7.7%) | 11 (5.3%) |  |
|  | | | | | |

### Supplemental Table 9: Description of treatment according frailty in NTE patients (N=832)

|  | | **NTE patients** | | **p** |
| --- | --- | --- | --- | --- |
|  | | **Non-Frail (N=284)** | **Frail (N=548)** |  |
| **Combinations. n (%)** | bortezomib; lenalidomide +/- corticoids (VRD) | 138 (48.6%) | 86 (15.7%) |  |
|  | lenalidomide +/- corticoids (RD) | 40 (14.1%) | 146 (26.6%) |  |
|  | bortezomib; melphalan +/- corticoids (MPV) | 49 (17.3%) | 133 (24.3%) |  |
|  | bortezomib; cyclophosphamide +/- corticoids (VCD) | 24 (8.5%) | 75 (13.7%) |  |
|  | bortezomib +/- corticoids (VD) | 6 (2.1%) | 56 (10.2%) |  |
|  | bortezomib; thalidomide +/- corticoids (VTD) | 16 (5.6%) | 8 (1.5%) |  |
|  | bortezomib; cyclophosphamide; lenalidomide +/- corticoids | 2 (0.7%) | 6 (1.1%) |  |
|  | cyclophosphamide +/- corticoids (CP) | 0 (0.0%) | 7 (1.3%) |  |
|  | melphalan +/- corticoids (MP) | 1 (0.4%) | 5 (0.9%) |  |
|  | daratumumab; lenalidomide +/- corticoids (DRD) | 1 (0.4%) | 4 (0.7%) |  |
|  | melphalan; thalidomide +/- corticoids (MPT) | 1 (0.4%) | 4 (0.7%) |  |
|  | bortezomib; daratumumab; lenalidomide +/- corticoids | 2 (0.7%) | 1 (0.2%) |  |
|  | bortezomib; doxorubicin +/- corticoids | 0 (0.0%) | 3 (0.5%) |  |
|  | bendamustine +/- corticoids (BP) | 0 (0.0%) | 2 (0.4%) |  |
|  | bendamustine; melphalan +/- corticoids | 0 (0.0%) | 2 (0.4%) |  |
|  | Other | 4 (1.6%) | 10 (2.0%) |  |
|  | | | | |
| **Proteasome inhibitor. n (%)** |  | **240 (84.5%)** | **374 (68.2%)** | <0.0001 |
|  | bortezomib | 240 (84.5%) | 373 (68.1%) |  |
|  |  |  |  |  |
| **IMID. n (%)** |  | **203 (71.5%)** | **262 (47.8%)** | <0.0001 |
|  | lenalidomide | 185 (65.1%) | 247 (45.1%) |  |
|  |  |  |  |  |
| **Anti-CD38. n (%)** |  | **4 (1.4%)** | **9 (1.6%)** | 0.8 |
| **Alkylant. n (%)** |  | **79 (27.8%)** | **238 (43.4%)** |  |
|  | Melphalan | 53 (18.7%) | 146 (26.6%) |  |
|  | Cyclophosphamide | 27 (9.5%) | 89 (16.2%) |  |
|  | Others | 0 (0.0%) | 15 (2.7%) |  |
|  | | | | |
| **Category. n (%)** | anti-CD38 +/- others | 4 (1.4%) | 9 (1.6%) | <0.0001 |
|  | IMID + PI +/- others | 158 (55.6%) | 104 (19.0%) |  |
|  | IMID +/- others | 41 (14.4%) | 152 (27.7%) |  |
|  | PI +/- others | 80 (28.2%) | 267 (48.7%) |  |
|  | others | 1 (0.4%) | 16 (2.9%) |  |
|  | | | | |

### Supplemental Table 10: Description of treatment according to high risk (HR) and non-high-risk status in ASCT patients (N=1036)

|  | | **NTE** | | **p** |
| --- | --- | --- | --- | --- |
|  | | **non HR* (N=959)** | **HR (N=77)** |  |
| **Combinations. n (%)** | bortezomib; lenalidomide +/- corticoids (VRD) | 271 (28.3%) | 23 (29.9%) |  |
|  | bortezomib; melphalan +/- corticoids (MPV / VMD / VMP) | 207 (21.6%) | 20 (26.0%) |  |
|  | lenalidomide +/- corticoids (RD) | 203 (21.2%) | 16 (20.8%) |  |
|  | bortezomib; cyclophosphamide +/- corticoids (CBP / CBD / VCD) | 118 (12.3%) | 5 (6.5%) |  |
|  | bortezomib +/- corticoids (VD) | 72 (7.5%) | 3 (3.9%) |  |
|  | bortezomib; thalidomide +/- corticoids (VTD) | 35 (3.6%) | 3 (3.9%) |  |
|  | bortezomib; cyclophosphamide; lenalidomide +/- corticoids | 7 (0.7%) | 3 (3.9%) |  |
|  | cyclophosphamide +/- corticoids (CP) | 7 (0.7%) | 0 (0.0%) |  |
|  | melphalan +/- corticoids (MP) | 6 (0.6%) | 0 (0.0%) |  |
|  | daratumumab; lenalidomide +/- corticoids (DRD) | 5 (0.5%) | 0 (0.0%) |  |
|  | melphalan; thalidomide +/- corticoids (MPT) | 5 (0.5%) | 0 (0.0%) |  |
|  | bortezomib; daratumumab; lenalidomide +/- corticoids | 3 (0.3%) | 1 (1.3%) |  |
|  | bortezomib; doxorubicin +/- corticoids | 2 (0.2%) | 1 (1.3%) |  |
|  | bendamustine +/- corticoids (BP) | 2 (0.2%) | 0 (0.0%) |  |
|  | bendamustine; melphalan +/- corticoids | 2 (0.2%) | 0 (0.0%) |  |
|  | bortezomib; daratumumab +/- corticoids (DVD) | 2 (0.2%) | 0 (0.0%) |  |
|  | Other | 12 (1.3%) | 2 (2.6%) |  |
|  | | | | |
| **Proteasome inhibitor. n (%)** |  | 724 (75.5%) | 61 (79.2%) | 0.46 |
|  | Bortezomib | 723 (75.4%) | 61 (79.2%) |  |
|  | Carfilzomib | 1 (0.1%) | 0 (0.0%) |  |
| **IMID. n (%)** |  | 538 (56.1%) | 48 (62.3%) | 0.29 |
|  | Thalidomide | 41 (4.3%) | 4 (5.2%) |  |
|  | Lenalidomide | 495 (51.6%) | 44 (57.1%) |  |
|  | Pomalidomide | 4 (0.4%) | 0 (0.0%) |  |
| **Anti-CD38. n (%)** |  | 14 (1.5%) | 1 (1.3%) | 0.91 |
|  | Daratumumab | 13 (1.4%) | 1 (1.3%) |  |
|  | Isatuximab | 1 (0.1%) | 0 (0.0%) |  |
| **Alkylant. n (%)** | A | 359 (37.4%) | 30 (39.0%) |  |
|  | Melphalan | 223 (23.3%) | 22 (28.6%) |  |
|  | Cyclophosphamide | 134 (14.0%) | 8 (10.4%) |  |
|  | Bendamustine | 5 (0.5%) | 0 (0.0%) |  |
|  | Other cytotoxics | 8 (0.8%) | 2 (2.6%) |  |
|  | | | | |
| **Category. n (%)** | Anti-CD38 +/- others | 14 (1.5%) | 1 (1.3%) | 0.6 |
|  | IMID + PI +/- others | 318 (33.2%) | 31 (40.3%) |  |
|  | IMID +/- others | 210 (21.9%) | 16 (20.8%) |  |
|  | PI +/- others | 400 (41.7%) | 29 (37.7%) |  |
|  | Anti-CD38 +/- others | 17 (1.8%) | 0 (0.0%) |  |
|  | | | | |

*******includes non-HR patients and patients with an unknown cytogenetic feature (not done)*

### Supplemental Table 11: TTNT median and rates in A/ overall first line patients and B/ in patients with NDMM who received ASCT and in NTE patients with NDMM

**A/**

| **-** | **N = 1573** |
| --- | --- |
| **Median 95% [CI]** | 32.6 [29.1; 34.7] |
| **IQR** | [12.2; .] |
|  |  |
| **TTNT rate ([95% CI]. Kaplan Meier)** |  |
| **0 month** | 100 % [100; 100] |
| **6 months** | 83 % [81.1; 84.8] |
| **12 months** | 75.2 % [73.1; 77.4] |
| **18 months** | 67 % [64.5; 69.5] |
| **24 months** | 58.3 % [55.6; 61.1] |
| **30 months** | 51.9 % [49; 54.9] |
| **36 months** | 45.5 % [42.3; 48.6] |
| **42 months** | 41.6 % [38.3; 45] |
| **48 months** | 37.6 % [34; 41.2] |
| **54 months** | 33.1 % [28.2; 38.1] |
| **60 months** | . % [.; .] |

**B/**

| **Test Wilcoxon**  **p-value** <.0001 |  | **NDMM NTE**  **N = 1016** | **NDMM ASCT**  **N = 557** |
| --- | --- | --- | --- |
| **TTNT (95% [IC])** | **Median (95% [IC])** | 20.4 [18.3; 22.8] | 52.1 [48.4; .] |
|  | **IQR** | [5.6; 46.6] | [31; .] |
| **TTNT rate ([95% CI]. Kaplan Meier)** | |  |  |
| **6 months** | | 74.2 % [71.4; 76.8] | 98.9 % [97.6; 99.5] |
| **12 months** | | 64 % [60.9; 67] | 95.4 % [93.2; 96.9] |
| **18 months** | | 53.9 % [50.5; 57.1] | 90.1 % [87; 92.4] |
| **24 months** | | 44.6 % [41.1; 48.1] | 82.1 % [78.1; 85.4] |
| **30 months** | | 37.1 % [33.3; 40.8] | 76.8 % [72.2; 80.7] |
| **36 months** | | 31.6 % [27.7; 35.5] | 68.4 % [63.2; 73] |
| **42 months** | | 27.9 % [23.9; 32.1] | 63.7 % [58.1; 68.8] |
| **48 months** | | 24.5 % [20.3; 28.9] | 58.4 % [52; 64.2] |
| **54 months** | | 22.9 % [18.4; 27.6] | . % [.; .] |
| **60 months** | | . % [.; .] | . % [.; .] |
|  | |  |  |

### Supplemental Table 12: TTNT median and rates in A/ NTE patients who received R-based combination and B/ in NTE patients NTE patients who received non R-based combination

A/

| **-** | **N = 529** |
| --- | --- |
| TTNT (95% [IC]) | 30.3 [25.8; 37.7] |
| IQR | [9.4; .] |
|  |  |
| TTNT rate ([95% CI]. Kaplan Meier) |  |
| 0 months | 100 % [100; 100] |
| 6 months | 79.9 % [76.5; 83.3] |
| 12 months | 71.4 % [67.5; 75.4] |
| 18 months | 63.4 % [58.9; 67.9] |
| 24 months | 56.9 % [52; 61.9] |
| 30 months | 50.8 % [45.3; 56.4] |
| 36 months | 43.8 % [37.5; 50.1] |
| 42 months | 38 % [31; 45] |
| 48 months | 33.2 % [25.7; 40.7] |
| 54 months | 33.2 % [25.7; 40.7] |
| 60 months | . % [.; .] |

B/

| **-** | **N = 487** |
| --- | --- |
| TTNT (95% [IC]) | 14.9 [13; 17.3] |
| IQR | [4.3; 31.1] |
|  |  |
| TTNT rate ([95% CI]. Kaplan Meier) |  |
| 0 months | 100 % [100; 100] |
| 6 months | 67.9 % [63.8; 72.1] |
| 12 months | 56 % [51.5; 60.5] |
| 18 months | 44.5 % [39.8; 49.1] |
| 24 months | 33.3 % [28.7; 37.9] |
| 30 months | 25.4 % [20.9; 29.9] |
| 36 months | 21.4 % [16.9; 25.8] |
| 42 months | 19.4 % [14.9; 24] |
| 48 months | 17.2 % [12.5; 21.9] |
| 54 months | 14.4 % [8.9; 19.8] |
| 60 months | . % [.; .] |
